# Assessment of Lipid Peroxidation in Alzheimer’s Disease Differential Diagnosis and Prognosis

**DOI:** 10.3390/antiox11030551

**Published:** 2022-03-14

**Authors:** Laura Ferré-González, Carmen Peña-Bautista, Miguel Baquero, Consuelo Cháfer-Pericás

**Affiliations:** 1 Alzheimer’s Disease Research Group, Health Research Institute La Fe, 46026 Valencia, Spain; laufegon@alumni.uv.es (L.F.-G.); mariadelcarmen_pena@iislafe.es (C.P.-B.); 2 Division of Neurology, University and Polytechnic Hospital La Fe, 46026 Valencia, Spain; baquero_miq@gva.es

**Keywords:** Alzheimer’s disease, dementia, diagnosis, oxidative stress, lipid peroxidation, prognosis

## Abstract

Alzheimer’s disease (AD) and other dementias are becoming increasingly common in the older population, and the number of people affected is expected to increase in a few years. Nowadays, biomarkers used in early AD diagnosis are expensive and invasive. Therefore, this research field is growing. In fact, peroxidation by-products derived from the oxidation of brain lipids (arachidonic (AA), docosahexanoic (DHA) and adrenic acid (AdA)) could be potential biomarkers, participating in the mechanisms of inflammation, neurotoxicity and cell death in AD pathology. Previous studies have shown specificity between lipid peroxidation compounds and other dementias (e.g., Lewy bodies (DLB), frontotemporal dementia (FTD)), but more research is required. Lipid peroxidation compounds (prostaglandins, isoprostanes, isofurans, neuroprostanes, neurofurans, dihomo-isoprostanes and dihomo-isofurans) were analysed by liquid chromatography and mass spectrometry in plasma samples from participants classified into a healthy group (*n* = 80), a mild cognitive impairment due to AD group (*n* = 106), a mild dementia due to AD group (*n* = 70), an advanced dementia due to AD group (*n* = 11) and a group of other non-AD dementias (*n* = 20). Most of these compounds showed statistically significant differences between groups (*p* < 0.05), showing higher levels for the healthy and non-AD groups than the AD groups. Then, a multivariate analysis was carried out on these compounds, showing good diagnosis indexes (AUC 0.77, sensitivity 81.3%, positive predictive value 81%). Moreover, evaluating AD disease prognosis, two compounds (15-F_2t_-IsoP and 14(RS)-14-F_4t_-NeuroP) and three total parameters (isoprostanes, isofurans and neurofurans) showed significant differences among groups. Some compounds derived from the oxidation of AA, DHA and AdA have demonstrated their potential use in differential AD diagnosis. Specifically, 15-F_2t_-IsoP, 14(RS)-14-F_4t_-NeuroP and the total parameters for isoprostanes, isofurans and neurofurans have shown prognostic value for AD from its earliest stages to its most severe form.

## 1. Introduction

Alzheimer’s disease (AD) and other dementias are becoming increasingly common in the elderly population, with AD being the most prevalent [1]. According to the World Health Organisation, there are currently more than 55 million people in the world with dementia, and the number of people affected is expected to increase to 139 million by 2050 [2]. Early diagnosis is very important, so some AD biomarkers have been developed. In general, they are based on imaging techniques, and the determination of impaired proteins in the cerebrospinal fluid (CSF) [3,4]. These biomarkers have been increasingly accepted as diagnostic criteria for AD, but they are expensive and invasive.

Oxidative stress plays an important role in many neurodegenerative diseases, be lipid peroxidation being one of the main processes involved, due to the high lipid composition of the brain and its high oxygen consumption [5]. The peroxidation by-products from brain lipids include prostaglandins (PGs), isoprostanes (IsoPs), isofurans (IsoFs), neuroprostanes (NeuroPs), neurofurans (NeuroFs), dihomo-isoprostanes (dihomo-IsoPs) and dihomo-isofurans (dihomo-IsoFs), among others [6]. PGs, IsoPs and IsoFs are derived from the oxidation of arachidonic acid (AA), which is evenly distributed in grey and white matter; NeuroPs and NeuroFs are derived from the oxidation of docosahexanoic acid (DHA), mainly present in the grey matter of the brain; and dihomo-IsoPs and dihomo-IsoFs are derived from the oxidation of adrenic acid (AdA), mainly present in white matter [7]. Polyunsaturated fatty acids are oxidised under oxidative stress conditions, inducing intramolecular reorganisations in their structures [6,8], as well as contributing to inflammation, neurotoxicity and cell death mechanisms in AD pathology [9,10,11]. Specifically, excessive lipid oxidation changes the physical properties of cell membranes and can induce the covalent modification of proteins and nucleic acids to cause necrosis, apoptosis and, more specifically, oxidative stress [12,13].

The biological role of these peroxidised by-products has attracted much attention. In fact, their pathological mechanisms are associated with neurological disorders, and they could show practical clinical applications as biomarkers [14,15]. Since alterations in fatty acids and brain lipid peroxidation have been detected in the early stages of AD [16], lipids and derivates have become compounds of interest to find biomarkers for the prediction, diagnosis and prevention of this disease [17]. Multiple biomarkers of lipid peroxidation are differentially expressed in easily accessible biological fluids during AD progression. Recent studies have also shown an increase in these compounds in the post-mortem brain of subjects with mild cognitive impairment (MCI) and preclinical AD [18].

Regarding specificity between lipid peroxidation and AD [19], current evidence suggests that these blood biomarkers may also be important for other common neurodegenerative disorders, such as dementia with Lewy bodies (DLB) or frontotemporal dementia (FTD) [7,20]. In fact, FTD is characterised by brain atrophy in the frontal and temporal regions, the concomitant lipid loss and dyslipidemia. However, not much is known about global lipid changes and underlying lipid dysregulation. Several studies have demonstrated differences in global lipid analysis forFTD compared to controls and AD subjects [21,22]. In the case of DLB, one study observed similarities when fatty acids were quantified in subjects with moderate AD and DLB [23]. Therefore, the aim of this work is to evaluate the possibility of using blood lipid peroxidation compounds to make a differential diagnosis between dementias due to AD and non-AD, as well as to see the progression of AD from MCI to the dementia stage.

## 2. Materials and Methods

### 2.1. Participants

This study was carried out in the Neurology Unit of the University and Polytechnic Hospital La Fe, Valencia (Spain). The sample included 287 participants aged between 45 and 80 years old; they were classified into a healthy group (*n* = 80), MCI due to AD group (MCI-AD, *n* = 106), mild dementia due to AD group (MD-AD, *n* = 70), advanced dementia due to AD group (AD-AD, *n* = 11) and dementias not due to AD group (non-AD, *n* = 20). The latter group consisted of patients diagnosed with FTD or DLB. It should be noted that the study protocol was approved (approve number: 2019/0105) by the Ethics Committee (CEIC) and all the participants gave their prior consent.

The participants’ classification was accomplished according to a neuropsychological evaluation (AD Co-operative Study ADL Scale for Mild Cognitive Impairment (ADCS-ADL-MCI) [24], Clinical Dementia Rating (CDR) [25], Functionality Assessment Questionnaire (FAQ) [26], Mini-Mental State Examination (MMSE) [27], Repeatable Battery for the Assessment of Neuropsychological Status-Delayed Memory (RBANS.DM) [28]) and CSF biomarkers (ß-amyloid-42, total tau (t-tau) and phosphorylated tau (p-tau)), following the recommendations of the National Institute on Aging-Alzheimer’s Association (NIA-AA) [29].

Table 1 shows the criteria used to classify patients into their respective groups. Therefore, the healthy group included participants with negative levels for CSF AD biomarkers (β-amyloid-42 > 725 pg·mL^−1^, t-tau < 485 pg·mL^−1^, p-tau < 56 pg·mL^−1^) and normal cognitive tests (ADCS-ADL-MCI > 44, CDR = 0, FAQ < 9, MMSE ≥ 27, RBANS.DM ≥ 85). The MCI-AD group involved positive CSF AD biomarkers (β-amyloid-42 < 725 pg·mL^−1^, t-tau > 485 pg·mL^−1^, p-tau > 56 pg·mL^−1^) and mild cognitive impairment (ADCS-ADL-MCI < 44, CDR ≤ 0.5, FAQ < 9, MMSE ≤ 27, RBANS.DM ≤ 85). The MD-AD group involved positive CSF AD biomarkers (β-amyloid-42 < 725 pg·mL^−1^, t-tau > 485 pg·mL^−1^, p-tau > 56 pg·mL^−1^) and cognitive impairment (ADCS-ADL-MCI < 44, CDR ≤ 1, FAQ > 9, MMSE ≤ 27, RBANS.DM ≤ 85). The AD-AD group involved positive CSF AD biomarkers (β-amyloid-42 < 725 pg·mL^−1^, t-tau > 485 pg·mL^−1^, p-tau > 56 pg·mL^−1^) and severe cognitive impairment (ADCS-ADL-MCI < 44, CDR ≥ 2, FAQ > 9, MMSE ≤ 27, RBANS.DM ≤ 85). The non-AD group included participants with negative CSF AD biomarkers (β-amyloid-42 > 725 pg·mL^−1^, t-tau < 485 pg·mL^−1^, p-tau < 56 pg·mL^−1^) and cognitive impairment based on at least one of these tests (ADCS-ADL-MCI < 44, CDR ≤ 1, FAQ > 9, MMSE ≤ 27, RBANS.DM ≤ 85).

### 2.2. Lipid Peroxidation Compounds Determination and Samples Analysis

The determined lipid peroxidation compounds were classified into several families. First, in the PGs family, four compounds (PGE_2_, PGF_2α_, 15-E_2t_-IsoP, 15-F_2t_-IsoP) were analysed. The family of IsoPs was composed of five compounds (15(R)-15-F_2t_-IsoP, 2,3-dinor-15-epi-15-F_2t_-IsoP, 15-keto-15-E_2t_-IsoP, 15-keto-15-F_2t_-IsoP, 5-F_2t_-IsoP) and one total parameter (total IsoPs). One total parameter was determined in the family of IsoFs. In the NeuroPs group, three compounds (4(RS)-4-F_4t_-NeuroP, 10-epi-10-F_4t_-NeuroP, 14(RS)-14-F_4t_-NeuroP) and one total parameter (total neuroPs) were analysed. Another total parameter was evaluated in the family of NeuroFs. In the dihomo-IsoPs group, four compounds were analysed (1a,1b-dihomo-PGF_2α_, Ent-7(RS)-7-F_2t_-dihomo-IsoP, 17-F_2t_-dihomo-IsoP, 17-epi-17-F_2t_-dihomo-IsoP). Finally, two lipids (17(RS)-10-epi-SC-Δ^15^-11-dihomo-IsoF, 7(RS)-ST-Δ^8^-11-dihomo-IsoF) were assessed in the dihomo-IsoFs group.

Blood samples from participants were collected between 8 and 10 a.m. following the established clinical procedures, and were centrifuged for 10 min at 2000× *g*; the plasma fraction was separated in a new tube with butylated hydroxytoluene (BHT) as the antioxidant, and stored at −80 °C before analysis. The treatment and analysis of the samples were carried out as described by Peña-Bautista et al. [17].

### 2.3. Statistical Analysis

Univariant analysis was carried out using IBM Statistical Package for the Social Sciences software version 23.0 (SPSS, Inc., Chicago, IL, USA), and multivariant analysis was carried out using Unscrambler X 10.4 software (Oslo, Norway).

For descriptive analysis, categorical variables were expressed as frequencies and percentages (%), and numeric variables were expressed as medians and interquartile ranges (IQR). In all the cases, the statistical significance was set st a *p* value ≤ 0.05.

Furthermore, the differences among medians were analysed by using non-parametric tests (Kruskal–Wallis, Mann–Whitney U test). Multivariate analysis was based on Partial Least Squares (PLS) regression using the dependent variables lipid peroxidation compounds (PGE2, 15-E2t-IsoP, 15-keto-15-E2t-IsoP, 15-keto-15-F2t-IsoP, 5-F2t-IsoP, total IsoPs, total IsoFs, 4(RS)-4-F4t-NeuroP, 10-epi-10-F4t-NeuroP, 14(RS)-14-F4t-NeuroP, total NeuroPs, total NeuroFs, 1a,1b-dihomo-PGF2α, Ent-7(RS)-7-F2t-dihomo-IsoP), age and gender, and one independent variable (AD, non-AD). Then, the receiver operating characteristic curve (ROC) was developed from the PLS model.

## 3. Results

### 3.1. Patients’ Characteristics

The demographic and clinical characteristics of the participants are described in Table 2. The clinical variables were used to differentiate among the groups of participants. As can be observed, the CSF biomarkers levels (β-amyloid42, t-tau, p-tau) and the neuropsychological evaluation allowed for the differentiation of the AD from the non-AD participants, as well as the identification of the stages of the disease, respectively. Expectedly, the healthy and non-AD groups showed higher levels of β-amyloid-42 and lower levels of t-tau and p-tau than the AD groups. The AD and non-AD groups also showed some impaired scores on neuropsychological tests, especially in the AD groups with more advanced disease states.

### 3.2. Lipid Peroxidation Compounds

The concentrations of analytes found in plasma samples from the participants groups are summarised in Table 3. As can be observed, most compounds showed statistically significant differences among the groups; specifically, higher levels were obtained for the healthy and non-AD groups than for the AD groups. Furthermore, a comparative analysis of the lipid peroxidation compounds between the groups is presented in Table 4. In the PGs family, significant differences were observed among all the groups in the PGE_2_ (*p* = 0.03) and 15-E_2t_-IsoP (*p* < 0.01) compounds, and PGF_2α_ is almost significant (*p* = 0.058) (Table 3). As can be seen in Table 4, significant differences were found for PGE_2_ between the healthy and MCI AD groups (*p* < 0.01), and between the MCI-AD and MD-AD groups (*p* = 0.05). In the case of PGF_2α_, significant differences were found between the non-AD group and the healthy (*p* < 0.01) and MCI (*p* < 0.01) groups. 15-E_2t_-IsoP also showed differences when comparing the healthy group with all the other AD groups (*p* < 0.01), and 15-F_2t_-IsoP only showed differences between MCI-AD and MD-AD (*p* = 0.01).

For IsoPs, significant differences were found among all participant groups for most of these compounds (15-keto-15-E_2t_-IsoP, 15-keto-15-F_2t_-IsoP, 5-F_2t_-IsoP and total IsoPs; *p* < 0.01; Table 3). Specifically, there are significant differences for 15-keto-15-E_2t_-IsoP between the healthy and the MCI-AD (*p* < 0.01), MD-AD (*p* < 0.01) and AD-AD (*p* = 0.03) groups. The lipid 15-keto-15-F_2t_-IsoP showed differences between the MCI-AD group and the healthy (*p* < 0.01), MD-AD (*p* = 0.02) and non-AD (*p* < 0.01) groups. In the case of 5-F_2t_-IsoP, differences were found between the healthy and the MCI-AD (*p* < 0.01) and AD-AD (*p* = 0.01) groups, as well as between the non-AD and the MCI-AD (*p* < 0.01) or MD-AD (*p* = 0.01) groups. In addition, total IsoPs showed significant differences between the healthy group and the MCI-AD (*p* < 0.01), MD-AD (*p* = 0.01) and AD-AD (*p* < 0.01) groups. Furthermore, differences were found between the MCI-AD and the non-AD (*p* = 0.01) and AD-AD (*p* = 0.02) groups, as well as between the MD-AD and AD-AD (*p* < 0.01) groups (Table 4). Regarding confounding variables, no significant differences were observed between the lipid peroxidation levels and sex.

Regarding IsoFs, total IsoFs showed significant differences among all the groups (*p* < 0.01; Table 3). Specifically, differences were observed between the healthy group and the MCI-AD (*p* < 0.01), and AD-AD (*p* < 0.01) groups, and there were almost significant differences with the MD-AD group (*p* = 0.051). Furthermore, differences were found between the MCI-AD and non-AD (*p* = 0.02) and AD-AD (*p* = 0.01) groups, as well as between the MD-AD and AD-AD (*p* < 0.01) groups (Table 4).

Regarding NeuroPs, significant differences were observed among all groups (*p* < 0.01). As we can see in Table 4, 4(RS)-4-F_4t_-NeuroP showed differences comparing the healthy group with all the other AD groups (*p* < 0.05). For 10-epi-10-F_4t_-NeuroP, differences were observed between the non-AD group and the healthy, MCI-AD and MD-AD (*p* < 0.01) groups. In the case of 14(RS)-14-F_4t_-NeuroP, differences were found between the AD-AD and the healthy and MD-AD groups (*p* < 0.01), and there were almost significant differences between the healthy and MCI-AD group (*p* = 0.059). Total NeuroPs also showed significant differences between the healthy group and the MCI-AD and MD-AD groups (*p* < 0.01).

Regarding NeuroFs, specifically total NeuroFs, significant differences were observed between the AD-AD and the healthy (*p* < 0.01), MCI-AD (*p* < 0.01) and MD-AD (*p* = 0.02) groups; as well as non-AD compared with healthy (*p* = 0.04);there were almost significant differences with the MCI-AD group (*p* = 0.054).

Within dihomo-IsoPs, significant differences were found among all groups for 1a,1b-dihomo-PGF_2__α_ and Ent-7(RS)-7-F_2t_-dihomo-IsoP (*p* < 0.01); they were almost significant for 17-epi-17-F_2t_-dihomo-IsoP (*p* = 0.054). As can be observed in Table 4, 1a,1b-dihomo-PGF_2__α_ showed significant differences between the healthy group and all AD groups (*p* < 0.01). Furthermore, significant differences were observed between the non-AD group and the MCI-AD and MD-AD groups (*p* < 0.01). In the case of Ent-7(RS)-7-F_2t_-dihomo-IsoP, there were differences between the healthy and the MCI-AD (*p* = 0.01), MD-AD (*p* < 0.01) and non-AD (*p* = 0.02) groups, as well as between the MCI-AD and MD-AD groups (*p* = 0.01). For 17-F_2t_-dihomo-IsoP, only differences between the healthy and MD-AD groups (*p* = 0.04) were observed. In addition, differences were observed in levels of 17-epi-17-F_2t_-dihomo-IsoP between the healthy and the MCI-AD and MD-AD groups (*p* < 0.01).

In the dihomo-IsoFs group,17(RS)-10-epi-SC-Δ^15^-11-dihomo-IsoF in particular showed differences between the non-AD and the healthy (*p* = 0.02) and MCI-AD (*p* = 0.04) groups, and almost showed differences between the healthy and MD-AD groups (*p* = 0.059).

**Figure 1:**
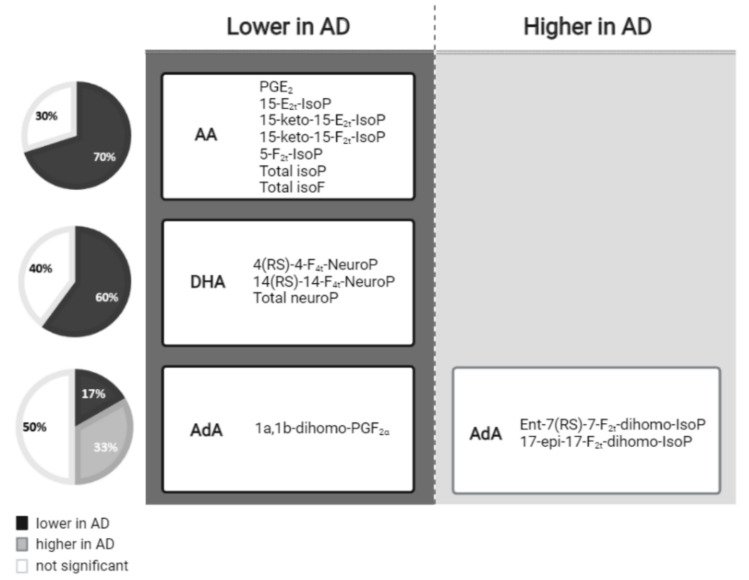
Lipid peroxidation compounds grouped by the fatty acid from which they were derived, and classified according to their levels in the AD versus non-AD groups.

In summary, as can be seen in Figure 1, most AA-derived compounds, DHA and one AdA-derived compound showed lower levels in AD, while two AdA-derived compounds showed higher levels in AD.

### 3.3. Multivariate Analysis

**Figure 2:**
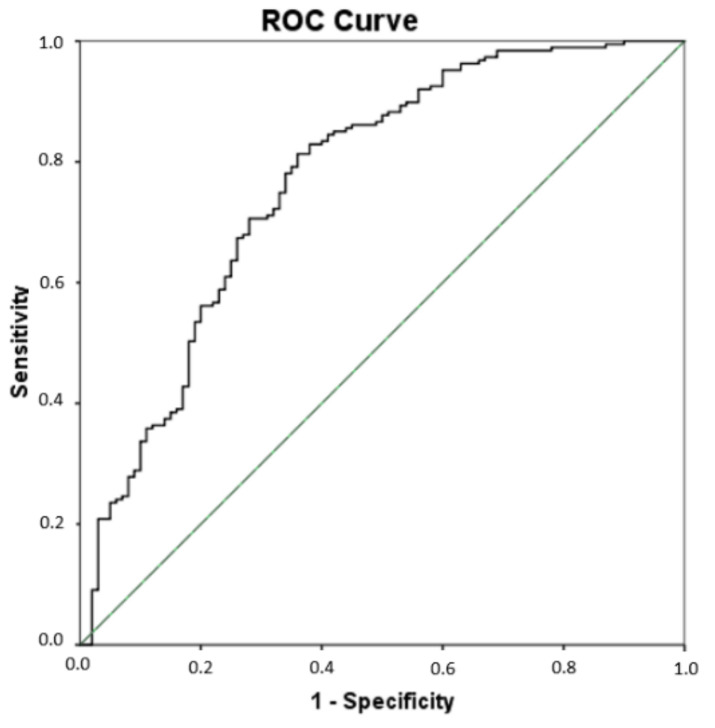
Receiver operating characteristic (ROC) curve for the differential diagnosis of AD developed from the multivariate PLS model. The area under curve (AUC) is 0.77 (95% Confidence interval (CI), 0.71–0.83).

Since significant differences were found for some lipid peroxidation compounds (PGE_2_, 15-E_2t_-IsoP, 15-keto-15-E_2t_-IsoP, 15-keto-15-F_2t_-IsoP, 5-F_2t_-IsoP, total IsoPs, total IsoFs, 4(RS)-4-F_4t_-NeuroP, 10-epi-10-F_4t_-NeuroP, 14(RS)-14-F_4t_-NeuroP, total NeuroPs, total NeuroFs, 1a,1b-dihomo-PGF_2__α_, Ent-7(RS)-7-F_2t_-dihomo-IsoP) among all groups of participants, a multivariate PLS analysis was carried out that included all these compounds simultaneously, to assess their utility in the AD differential diagnosis. As result, the sensitivity was 81.3%, the specificity was 64%, the accuracy was 75.3%, the positive predictive value was 81% and the odds ratio was 7.72 (see Table 5). Figure 2 depicts the corresponding ROC curve developed from the PLS model. The corresponding cut-off value was 0.619.

### 3.4. Alzheimer’s Disease Prognosis

In the prognosis evaluation of the disease, the AD groups (MCI-AD, MD-AD, AD-AD) were studied. As can be observed in Table 6, compound 15-F_2t_-IsoP, total IsoPs, total IsoFs, compound 14(RS)-14-F_4t_-NeuroP and total NeuroFs showed significant differences among groups (*p* < 0.05). Regarding the trend they follow, it can be seen in Table 3. In fact, the highest levels for the NeuroFs total parameter were found for the MCI-AD group (MCI > MD > AD), but for the other compounds, the highest levels were found in the MD-AD group (MD > MCI > AD).

## 4. Discussion

In this work, 18 lipid peroxidation compounds and 4 total parameters were quantified simultaneously in plasma samples from 5 biologically defined participant groups. Some of these biomarkers showed statistically significant differences among the groups.

First, PGs, IsoPs and IsoFs come from AA oxidation. However, PGs are inflammatory molecules, which are formed following the cyclooxygenase metabolic pathway and are associated with cognitive impairment [6,30].

In the PGs peroxidation compounds, differences in PGE_2_ and 15-E_2t_-IsoP were observed between all the participant groups. Specifically, patients with AD showed lower levels compared with the healthy group, in contrast with other studies carried out on CSF post-mortem or urine samples [6,31]. Nevertheless, levels decreased with disease progression in the case of PGE_2_ [31]. This may be explained by the fact that some metabolites continue their oxidation of other compounds of lower molecular weight throughout the course of the disease [32]. In other non-AD diseases such as Creutzfeldt-Jakob disease, cases had higher levels of PGs than healthy people [33,34]. However, in cases of FTD and DLB diseases, no significant differences were found between the healthy and the case groups in terms of AA oxidation [23], whereas in our study, non-AD patients showed significantly lower levels compared with the healthy group. Regarding IsoPs and IsoFs, they are prostaglandin-like compounds which are formed via the non-enzymatic, but free radical, catalysed peroxidation of AA through the activation of cytosolic phospholipases [6,33,35]. IsoFs are preferentially formed instead of IsoP in situations of increased oxygen tension [36]. Therefore, observing their formation helps to better define the involvement of oxidative stress in many neurological diseases [33]. Among these molecules, significant differences were noticed between all participant groups. In all of them, the same trend as in the PGs was observed. In fact, AD patients showed lower levels compared with the healthy group, in contrast with previous studies performed on brain tissue [19,37], CSF [6] and urine [31], but corroborating previous results observed in another study and realised in plasma samples [38]. However, these levels rose as AD progressed (MCI-AD < MD-AD), as expected from a previous study [39]. Nevertheless, declines in these levels were observed in more advanced stages of the disease, but could not be representative due to the small sample size of the AD-AD group in the present study. For non-AD patients, several studies did not find significant differences for IsoPs [19,40,41]. However, in this work, levels of 15-keto-15-E_2t_-IsoP, total IsoPs and total IsoFs in the non-AD group were lower than the healthy group and higher than the AD groups. Moreover, 15-keto-15-F_2t_-IsoP and 5-F_2t_-IsoP levels were higher in the non-AD than the healthy and AD groups, as observed in other studies carried out on brain tissue [23,42], CSF [6] and urine [41].

Second, NeuroPs and NeuroFs are formed by a free radical non-enzymatic mechanism involving the peroxidation of DHA [43], and are considered robust in vivo biomarkers of oxidative stress in diseases [44]. For all the NeuroPs compounds, AD patients showed lower levels compared with the healthy group, except for 10-epi-10-F_4t_-NeuroP, while other studies did not find significant increases in CSF samples for AD patients compared to controls [38,45]. Other plasma and urine studies from the literature claimed that NeuroPs did not reflect differences between groups [46]. Furthermore, higher levels were found in brain tissue from patients with advanced stages of AD than in MCI-AD [47]. Lower levels of NeuroPs were also obtained in non-AD patients than in healthy patients, but were higher than the AD groups, while other studies reported an increase in other neurodegenerative diseases [6]. Regarding NeuroFs compounds, patients with AD showed lower levels compared with the healthy group, in contrast with a previous study, which showed an elevation of these levels in the cerebral cortex of a mouse AD model [48]. In addition, NeuroFs levels decreased with disease progression, as corroborated in a previous study carried out on urine samples [32], as well as in another study carried out on brain tissue [23]. Also, non-AD patients showed lower levels compared with the healthy and AD groups, contrary to another study carried out on brain tissue [23].

Third, dihomo-IsoPs are the peroxidation products from AdA, which is the major component of white matter. These compounds could be used as selective in vivo quantitative biomarkers of free radical damage to neuronal membranes [6,49,50]. Specifically, 1a,1b-dihomo-PGF_2α_ showed the highest levels for the non-AD group, corroborating previous studies carried out on urine [51] and plasma [52], which reported higher levels in the non-AD neurological disorders group compared to the controls. In addition, the Ent-7(RS)-7-F_2t_-dihomo-IsoP compound exhibited higher levels in patients with dementia due to AD than healthy or non-AD patients, similarly to studies determining the total lipid fraction of various brain regions (hippocampus), which showed increased AdA content in AD [53]. However, this contrasted with another study, which showed higher levels in the control group compared to the AD group [54].

Focusing on the AD groups and prognosis evaluation, two lipid peroxidation compounds (15-F_2t_-IsoP and 14(RS)-14-F_4t_-NeuroP) and three total parameters (total IsoPs, total IsoFs, total NeuroFs) showed a prognostic value for the disease. Interestingly, most of these compounds showed their highest levels in the MD-AD group compared to the MCI-AD and AD-AD groups (MD > MCI > AD). Actually, a tendency for lipid peroxidation compounds to increase as AD developmed was observed. Nevertheless, the group with the most advanced stage of the disease (AD-AD) could not be representative due to its small sample size compared to the other groups. However, total NeuroFs showed the highest levels in the MCI-AD group compared to the MD-AD and AD-AD groups (MCI > MD > AD). In contrast to other studies [39,55], it was observed that these lipid peroxidation metabolites decreased as AD progressed, which may be explained by the oxidation of some metabolite-generating compounds of lower molecular weight throughout the course of AD [32].

As can be seen, there is both agreement and discrepancy with other studies regarding lipid peroxidation levels in AD progression and in other types of dementia. These differences could be due to the different type of sample used for the analysis, so more research in this field is required to compare lipid peroxidation compounds levels in well-defined patients and in different types of samples.

Among the study’s limitations, these results use an approximation of AD progression since it is not a prospective study, although it does include patients at different stages of disease progression. Furthermore, the small sample size of the AD-AD and non-AD groups compared to the other groups is evident, and could lead to a reduction in statistical power. In addition, other confounding variables such as the participants’ diet or use of anti-hyperlipidemic drugs, which could interfere with the blood lipid peroxidation levels, has not been considered.

## 5. Conclusions

The measurements of lipid peroxidation compounds have been used to elucidate the role of oxidative stress in neurodegenerative disease conditions. Specifically, these compounds, derived from AA, DHA and AdA oxidation, could be considered promising plasma biomarkers for early and differential AD diagnosis. In addition, among the evaluated analytes, 15-F_2t_-IsoP, 14(RS)-14-F_4t_-NeuroP and the total parameters of IsoPs, IsoFs and NeuroFs have shown prognostic value for AD from the earliest stages to the most severe form. Nevertheless, the great controversy with regard to previous studies makes it necessary to carry out further research validating these potential biomarkers in large and well-defined cohorts.

## Figures and Tables

**Table 1:** Participants’ classification criteria, attending to CSF biomarkers and neuropsychological assessment.

Clinical Evaluation	Classification of Participants				
	Healthy	MCI-AD	MD-AD	AD-AD	Non-AD
β-Amyloid-42 (pg·mL^−1^)	>725	<725	<725	<725	>725
t-tau (pg·mL^−1^)	<485	>485	>485	>485	<485
p-tau (pg·mL^−1^)	<56	>56	>56	>56	<56
ADCS-ADL-MCI	>44	<44	<44	<44	<44
CDR	0	≤0.5	≤1	≥2	≤1
FAQ	<9	<9	>9	>9	>9
MMSE	≥27	≤27	≤27	≤27	≤27
RBANS.DM	≥85	≤85	≤85	≤85	≤85

t-tau: total tau; p-tau: phosphorylated tau; ADCS-ADL-MACI: AD Co-operative Study ADL Scale for Mild Cognitive Impairment; CDR: Clinical Dementia Rating; FAQ: Functionality Assessment Questionnaire; MMSE: Mini-Mental State Examination; RBANS, Repeatable Battery for the Assessment of Neuropsychological Status (DM: delayed memory; A: attention; L: learning; VC: visuospatial/constructional; IM: immediate memory).

**Table 2:** Demographic and clinical characteristics of the participants.

Variable		Healthy (*n* = 80)	MCI-AD (*n* = 106)	MD-AD (*n* = 70)	AD-AD (*n* = 11)	Non-AD (*n* = 20)	*p* Value (Kruskal-Wallis Test)
Demographic characteristics							
Age (years, median (IQR))		63 (61–68)	70 (67–74)	71 (67–74)	66 (70–73)	65 (59–71)	<0.01
Gender (female (%))		44 (55%)	61 (57.5%)	46 (65.7%)	7 (63.6%)	11 (55%)	0.70
Level of education *n* (%)	Basic Secondary University	22 (27.5%) 21 (26.3%) 37 (46.3%)	59 (55.7%) 23 (21.7%) 24 (22.6%)	41 (58.6%) 16 (22.9%) 13 (18.6%)	9 (81.8%) 1 (9.1%) 1 (9.1%)	14 (70%) 3 (15%) 3 (15%)	<0.01
Clinical characteristics							
β-Amyloid-42 (pg·mL^−1^, median (IQR))		1201 (942.75–1439.75)	597 (471.05–709.07)	574 (444.75–648.50)	636 (601.76–708.88)	982 (858–1647)	<0.01
t-tau (pg·mL^−1^, median (IQR))		224 (173.25–304)	583 (432.50–773.50)	621 (448.50–945.50)	699 (473–936)	289 (205–376)	<0.01
p-tau (pg·mL^−1^, median (IQR))		36 (28–47.25)	90 (70.50–109.50)	88.50 (73–144)	95 (73–122)	40 (32–61)	<0.01
ADCS-ADL-MCI (median (IQR))		47 (43–50.75)	41 (31–46)	36 (24–42)	29.50 (3–34.50)	29 (22.50–40.75)	<0.01
CDR (median (IQR))		0 (0–0)	0.5 (0.5–0.5)	1 (0.5–1)	2 (2–3)	1 (0.5–1)	<0.01
FAQ (median (IQR))		0 (0–2)	4 (1–7)	13 (10–17.25)	21 (14–25)	11.50 (8–21)	<0.01
MMSE (median (IQR))		29 (28–30)	25 (22–27.25)	20 (17.75–24)	14 (12–19)	23 (18–25)	<0.01
RBANS.DM (median (IQR))		100 (95–106)	52 (40–75)	40 (40–49)	40 (40–44)	58 (45–78)	<0.01

t-tau: total tau; p-tau: phosphorylated tau; ADCS-ADL-MACI: AD Co-operative Study ADL Scale for Mild Cognitive Impairment; CDR: Clinical Dementia Rating; FAQ: Functionality Assessment Questionnaire; MMSE: Mini-Mental State Examination; RBANS, Repeatable Battery for the Assessment of Neuropsychological Status (DM: delayed memory; A: attention; L: learning; VC: visuospatial/constructional; IM: immediate memory).

**Table 3:** Levels of lipid peroxidation compounds in plasma samples from each participants group.

Variable (nmol·L^−1^)		Healthy (*n* = 80) Median (1st, 3rd Quartile)	MCI-AD (*n* = 106) Median (1st, 3rd Quartile)	MD-AD (*n* = 70) Median (1st, 3rd Quartile)	AD-AD (*n* = 11) Median (1st, 3rd Quartile)	Non-AD (*n* = 20) Median (1st, 3rd Quartile)	*p* Value (Kruskal-Wallis Test)
PGs ^a^	PGE_2_	0.27 (0.05–0.45)	0.07 (0–0.35)	0.12 (0.04–0.50)	0.10 (0–0.20)	0.10 (0.07–0.16)	0.03 *
	PGF2_α_	0.55 (0.28–0.86)	0.60 (0.10–0.85)	0.63 (0.26–1.13)	0.60 (0.30–0.80)	0.88 (0.53–1.38)	0.058 ^
	15-E_2t_-IsoP	0.78 (0.27–1.56)	0.31 (0.09–1.01)	0.30 (0.07–1.08)	0.25 (0–0.67)	0.13 (0.05–0.29)	<0.01 *
	15-F_2t_-IsoP	0.03 (0.01–0.06)	0.02 (0–0.05)	0.03 (0–0.21)	0.02 (0–0.03)	0 (0–0.57)	0.08
IsoPs ^a^	15(R)-15-F_2t_-IsoP	0.31 (0.20–0.59)	0.30 (0.16–0.53)	0.35 (0.20–0.58)	0.25 (0.06–0.45)	0.48 (0.21–0.75)	0.21
	2,3-dinor-15-epi-15-F_2t_-IsoP	0 (0–0.02)	0 (0–0)	0 (0–0)	0 (0–0)	0 (0–0)	0.58
	15-keto-15-E_2t_-IsoP	0.33 (0–0.92)	0 (0–0.17)	0.01 (0–0.25)	0 (0–0.32)	0.05 (0–0.38)	<0.01 *
	15-keto-15-F_2t_-IsoP	0.37 (0.17–0.67)	0.25 (0.07–0.40)	0.33 (0.15–0.56)	0.27 (0.07–0.40)	0.47 (0.30–0.78)	<0.01 *
	5-F_2t_-IsoP	1.38 (0.88–2.62)	0.98 (0.33–1.69)	1.25 (0.65–2.00)	0.65 (0.35–1.45)	1.98 (1.08–2.55)	<0.01 *
	Total IsoPs	0.72 (0.31–1.39)	0.39 (0.26–0.67)	0.45 (0.30–0.68)	0.27 (0.22–0.37)	0.62 (0.44–0.79)	<0.01 *
IsoFs ^a^	Total IsoFs	0.33 (0.08–0.46)	0.14 (0.08–0.32)	0.19 (0.09–0.36)	0.08 (0.05–0.14)	0.23 (0.19–0.40)	<0.01 *
NeuroPs ^b^	4(RS)-4-F_4t_-NeuroP	1.37 (0.95–3.68)	1.10 (0–1.50)	1.08 (0.58–1.40)	0.97 (0–1.27)	1.03 (0.61–1.46)	<0.01 *
	10-epi-10-F_4t_-NeuroP	0.15 (0.06–0.22)	0.13 (0.01–0.22)	0.20 (0.05–0.32)	0.11 (0.07–0.20)	0.32 (0.25–0.41)	<0.01 *
	14(RS)-14-F_4t_-NeuroP	1.08 (0.13–1.89)	0.53 (0–1.58)	0.88 (0.25–1.30)	0.15 (0–0.42)	0.91 (0.60–1.43)	0.01 *
	Total NeuroPs	0.05 (0–0.56)	0 (0–0.11)	0 (0–0.05)	0 (0–0.38)	0.01 (0–0.06)	<0.01 *
NeuroFs ^b^	Total NeuroFs	0.16 (0.06–0.32)	0.15 (0.05–0.33)	0.12 (0.05–0.25)	0.09 (0–0.11)	0.08 (0.04–0.15)	0.02 *
Dihomo-IsoPs ^c^	1a,1b-dihomo-PGF_2α_	0 (0–3.34)	0 (0–0)	0 (0–1.03)	0 (0–0)	1.18 (0–1.40)	<0.01 *
	Ent-7(RS)-7-F_2t_-dihomo-IsoP	0.03 (0–0.17)	0.10 (0–0.22)	0.15 (0.10–0.22)	0.15 (0.05–0.22)	0.12 (0.10–0.19)	<0.01 *
	17-F_2t_-dihomo-IsoP	0 (0–0)	0 (0–0)	0 (0–0)	0 (0–0)	0 (0–0)	0.10
	17-epi-17-F_2t_-dihomo-IsoP	0 (0–0)	0 (0–0.02)	0 (0–0.02)	0 (0–0.02)	0 (0–0)	0.054 ^
Dihomo-IsoFs ^c^	17(RS)-10-epi-SC-Δ^15^-11-dihomo-IsoF	0 (0–0)	0 (0–0)	0 (0–0)	0 (0–0)	0 (0–0)	0.07
	7(RS)-ST-Δ^8^-11-dihomo-IsoF	0 (0–0.22)	0 (0–0.17)	0.02 (0–0.10)	0.05 (0.02–0.12)	0 (0–0.09)	0.52

^a^ = Lipid peroxidation compounds derived from the oxidation of AA; ^b^ = Lipids derived from the oxidation of DHA; ^c^ = Lipids derived from the oxidation of AdA; * *p* value < 0.05; ^ *p* value near to 0.05.

**Table 4:** Comparison of lipid peroxidation compounds between groups using Mann–Whitney test.

Lipids		Healthy vs. MCI-AD	Healthy vs. MD-AD	Healthy vs. AD-AD	Healthy vs. Non-AD	MCI-AD vs. MD-AD	MCI-AD vs. AD-AD	MCI-AD vs. Non-AD	MD-AD vs. AD-AD	MD-AD vs. Non-AD
PGs ^a^	PGE_2_	<0.01 *	0.38	0.06	0.23	0.05 *	0.78	0.23	0.23	0.90
	PGF_2α_	0.66	0.30	0.97	<0.01 *	0.16	0.79	<0.01 *	0.50	0.09
	15-E_2t_-IsoP	<0.01 *	<0.01 *	0.01 *	<0.01 *	0.99	0.37	0.09	0.52	0.09
	15-F_2t_-IsoP	0.07	0.24	0.10	0.59	0.01 *	0.89	0.95	0.18	0.26
IsoPs ^a^	15(R)-15-F_2t_-IsoP	0.65	0.51	0.21	0.20	0.20	0.23	0.07	0.12	0.31
	2,3-dinor-15-epi-15-F_2t_-IsoP	0.48	0.19	0.40	0.22	0.46	0.61	0.40	0.91	0.73
	15-keto-15-E_2t_-IsoP	<0.01 *	<0.01 *	0.03 *	0.11	0.08	0.57	0.11	0.79	0.73
	15-keto-15-F_2t_-IsoP	<0.01 *	0.42	0.19	0.18	0.02 *	0.76	<0.01 *	0.35	0.05 *
	5-F_2t_-IsoP	<0.01 *	0.06	0.01 *	0.31	0.16	0.47	<0.01 *	0.10	0.01 *
	Total IsoPs	<0.01 *	0.01 *	<0.01 *	0.49	0.33	0.02 *	0.01 *	<0.01 *	0.09
IsoFs ^a^	Total IsoFs	<0.01 *	0.051 ^	<0.01 *	0.63	0.21	0.01 *	0.02 *	<0.01 *	0.18
NeuroPs ^b^	4(RS)-4-F_4t_-NeuroP	<0.01 *	<0.01 *	0.02 *	0.02 *	0.95	0.38	0.98	0.35	0.92
	10-epi-10-F_4t_-NeuroP	0.72	0.18	0.55	<0.01 *	0.10	0.73	<0.01 *	0.34	<0.01 *
	14(RS)-14-F_4t_-NeuroP	0.059 ^	0.26	<0.01 *	0.99	0.26	0.07	0.17	<0.01 *	0.49
	Total NeuroPs	<0.01 *	<0.01 *	0.15	0.28	0.73	0.98	0.34	0.98	0.12
NeuroFs ^b^	Total NeuroFs	0.73	0.39	<0.01 *	0.04 *	0.58	<0.01 *	0.054 ^	0.02 *	0.14
Dihomo-IsoPs ^c^	1a,1b-dihomo-PGF_2α_	<0.01 *	<0.01 *	<0.01 *	0.78	0.26	0.14	<0.01 *	0.06	<0.01 *
	Ent-7(RS)-7-F_2t_-dihomo-IsoP	0.01 *	<0.01 *	0.08	0.02 *	0.01 *	0.57	0.51	0.60	0.35
	17-F_2t_-dihomo-IsoP	0.56	0.04 *	0.51	0.06	0.08	0.42	0.13	0.21	0.82
	17-epi-17-F_2t_-dihomo-IsoP	<0.01 *	<0.01 *	0.26	0.48	0.65	0.82	0.36	0.68	0.27
Dihomo-IsoFs ^c^	17(RS)-10-epi-SC-Δ^15^-11-dihomo-IsoF	0.64	0.059 ^	0.59	0.02 *	0.09	0.51	0.04 *	0.27	0.53
	7(RS)-ST-Δ^8^-11-dihomo-IsoF	0.89	0.83	0.38	0.21	0.92	0.22	0.28	0.19	0.24

Notes: ^a^ = Lipid peroxidation compounds derived from the oxidation of AA; ^b^ = Lipids derived from the oxidation of DHA; ^c^ = Lipids derived from the oxidation of AdA; * *p* value < 0.05; ^ *p* value near to 0.05.

**Table 5:** Diagnosis indexes from the multivariate model of differential AD detection.

Parameter	(95% CI)
Sensitivity (%)	81.3 (75.1–86.2)
Specificity (%)	64 (54.2–72.7)
Accuracy (%)	75.3 (70–80)
Positive predictive value (%)	81 (74.6–85.8)
Negative predictive value (%)	64.6 (54.8–73.4)
Odds ratio	7.72 (4.46–13.37)

**Table 6:** Lipid peroxidation compounds in AD prognosis evaluation.

Lipids		*p* Value (Kruskal-Wallis Test)
PGs ^a^	PGE2	0.11
	PGF_2α_	0.36
	15-E_2t_-IsoP	0.71
	15-F_2t_-IsoP	0.03 *
IsoPs ^a^	15(R)-15-F_2t_-IsoP	0.17
	2,3-dinor-15-epi-15-F_2t_-IsoP	0.71
	15-keto-15-E_2t_-IsoP	0.22
	15-keto-15-F_2t_-IsoP	0.08
	5-F_2t_-IsoP	0.19
	Total IsoPs	0.02 *
IsoFs ^a^	Total IsoFs	0.01 *
NeuroPs ^b^	4(RS)-4-F_4t_-NeuroP	0.65
	10-epi-10-F_4t_-NeuroP	0.23
	14(RS)-14-F_4t_-NeuroP	0.03 *
	Total NeuroPs	0.94
NeuroFs ^b^	Total NeuroFs	0.03 *
Dihomo-IsoPs ^c^	1a,1b-dihomo-PGF2_α_	0.13
	Ent-7(RS)-7-F_2t_-dihomo-IsoP	0.06
	17-F_2t_-dihomo-IsoP	0.13
	17-epi-17-F_2t_-dihomo-IsoP	0.86
Dihomo-IsoFs ^c^	17(RS)-10-epi-SC-Δ^15^-11-dihomo-IsoF	0.15
	7(RS)-ST-Δ^8^-11-dihomo-IsoF	0.43

Notes: ^a^ = Lipids derived from the oxidation of AA; ^b^ = Lipids derived from the oxidation of DHA; ^c^ = Lipids derived from the oxidation of AdA; * *p* value < 0.05.

## Data Availability

The data presented in this study are available on request from m.consuelo.chafer@uv.es (C.C.-P.). The data are not publicly available for the protection of the individuals’ data.
